# The ArsH Protein Product of the *Paracoccus denitrificans ars* Operon Has an Activity of Organoarsenic Reductase and Is Regulated by a Redox-Responsive Repressor

**DOI:** 10.3390/antiox11050902

**Published:** 2022-05-03

**Authors:** Vojtěch Sedláček, Martin Kryl, Igor Kučera

**Affiliations:** Department of Biochemistry, Faculty of Science, Masaryk University, Kotlářská 2, 611 37 Brno, Czech Republic; 21931@seznam.cz (V.S.); 324471@mail.muni.cz (M.K.)

**Keywords:** FMN, NADPH, organoarsenicals, *Paracoccus denitrificans*, redox-responsive repressor

## Abstract

*Paracoccus denitrificans* ArsH is encoded by two identical genes located in two distinct putative arsenic resistance (*ars*) operons. *Escherichia coli*-produced recombinant N-His_6_-ArsH was characterized both structurally and kinetically. The X-ray structure of ArsH revealed a flavodoxin-like domain and motifs for the binding of flavin mononucleotide (FMN) and reduced nicotinamide adenine dinucleotide phosphate (NADPH). The protein catalyzed FMN reduction by NADPH via ternary complex mechanism. At a fixed saturating FMN concentration, it acted as an NADPH-dependent organoarsenic reductase displaying ping-pong kinetics. A 1:1 enzymatic reaction of phenylarsonic acid with the reduced form of FMN (FMNH_2_) and formation of phenylarsonous acid were observed. Growth experiments with *P. denitrificans* and *E. coli* revealed increased toxicity of phenylarsonic acid to cells expressing *arsH*, which may be related to in vivo conversion of pentavalent As to more toxic trivalent form. ArsH expression was upregulated not only by arsenite, but also by redox-active agents paraquat, tert-butyl hydroperoxide and diamide. A crucial role is played by the homodimeric transcriptional repressor ArsR, which was shown in in vitro experiments to monomerize and release from the DNA-target site. Collectively, our results establish ArsH as responsible for enhancement of organo-As(V) toxicity and demonstrate redox control of *ars* operon.

## 1. Introduction

*Paracoccus denitrificans*, a common soil bacterium, has become a model organism for studies on electron transfer and biological energy conservation [1]. Being a non-fermenting obligate respirer, aerobically growing organism has to cope with reactive oxygen species that are permanently formed as by-products of respiratory chain reactions. Our previous work has pointed to three nicotinamide adenine dinucleotide (phosphate) NAD(P)H-dependent oxidoreductases that are potentially involved in oxidative stress response/defense. The ferric reductase B (FerB), the product of the *pden_4071* gene, is present constitutively. It forms a homodimer containing one bound flavin mononucleotide (FMN) per monomer [2] and is enzymatically active in reduction of Fe(III)-ligand complexes [3], quinones [4,5], chromate [6] and superoxide [5,7]. The cellular sensitivity to oxidative stress is enhanced in FerB mutant [5].

By using a proteomic approach, we have identified two other similar proteins, Pden_5119 and Pden_3133, as being up-regulated under paraquat-mediated oxidative stress [8]. Pden_5119 is a flavin reductase preferring NADH rather than NADPH as the electron donor. The reduced flavin formed was found to remain associated with the catalytic site long enough to be back-oxidized by molecular oxygen, which is manifested by an activity of NADH oxidase. We also observed increased *pden_5119* transcript levels in cells stressed with methyl viologen, diamide or t-butyl hydroperoxide and a higher sensitivity of mutant cells to these agents compared to wild-type cells [9]. These results are consistent with a role for the Pden_5119 protein to maintain the cellular redox state.

Preliminary biochemical characterization of the *pden_3133* gene product showed that it is an NADPH-dependent FMN reductase [8]. Substrate specificity and primary structure homology suggested a close relationship with ArsH proteins. The *arsH* genes are present in some but not all *ars* operons encoding arsenic resistance/tolerance in bacteria [10]. ArsH proteins from different sources have been shown to catalyze the NADPH-dependent reduction of ferric iron [11,12], quinones [13], chromate [12], azo dyes [14,15,16] and a nitroaromatic antibiotic nitrofurazone [16]. The physiological role of ArsH in arsenic metabolism has long remained enigmatic until a study demonstrating that ArsH from *Pseudomonas putida*, rather than being a reductase, acts as a mixed-function oxidase, incorporating one oxygen atom from molecular oxygen to trivalent methylated and aromatic arsenicals to give less toxic pentavalent species [17]. Notwithstanding this possibility, however, more recent results indicate that the predominant activity of ArsH in *P. putida* may be the reduction of FMN by NADPH involved in quenching the oxidative stress caused by exposure to As salts or other redox stressors [18].

From the foregoing, it is evident that there are interesting connections to draw between arsenic resistance and oxidative stress defense provided by flavoproteins. In order to shed further light on these issues, the present study examines the altered cellular growth and *arsH* gene expression in *P. denitrificans* cells exposed to arsenicals or redox stressors and also the structural and enzymatic properties of the recombinant *P. denitrificans* ArsH protein heterologously produced in *Escherichia coli*. An effort was also devoted to identify *ars* repressor and operator and characterization of their interaction under in vitro conditions.

## 2. Materials and Methods

### 2.1. Bacterial Strains and Growth Conditions

The parental *Paracoccus denitrificans* strain Pd1222 was provided by Rob van Spanning (Vrije Universiteit Amsterdam, Amsterdam, The Netherlands). The basal growth succinate mineral medium for aerobic cultivation of *P. denitrificans* was composed of 17 mM NaH_2_PO_4_, 33 mM KH_2_PO_4_, 50 mM NH_4_Cl, 1 mM MgSO_4_, 30 μM ferric citrate and 50 mM succinate in distilled water adjusted to pH 7.3. The M9 medium for aerobic cultivation of *Escherichia coli* contained 42 mM Na_2_HPO_4_, 22 mM KH_2_PO_4_, 8.5 mM NaCl, 19 mM NH_4_Cl, 2 mM MgCl_2_, 0.1 mM CaCl_2_, 0.4% glucose and 0.01% thiamine. Pd1222 was prepared from glycerol stock grown overnight at 30 °C in brain heart infusion (BHI) medium supplemented with rifampicin at a concentration of 20 μg mL^−1^. An initial inoculum was made by transferring 0.2 mL of the BHI culture to 100 mL Erlenmeyer flask containing 15 mL of the succinate mineral medium and continuously agitating the flask for 12 h at 250 rpm and 30 °C on a closed orbital shaker KS15A (Edmund Buhler, GmbH, Bodelshausen, Germany). Growth curves for *P. denitrificans* and *E. coli* cultures were generated in triplicates at 30 °C in 200 μL volumes of the respective mineral medium in 96-well microplate formate using an ELx808 microplate reader (BioTek Instruments Inc., Winooski, VT, USA ). The wells were inoculated to an OD_600_ of 0.1 from the first inoculum and OD_600_ was automatically read at every 20 min for consecutive 48 h. The maximum specific growth rate *μ_max_* was calculated from growth curves fitted using a Gompertz-type model according to Equation (1) [19].

(1) $$ln\left(X_{t}\right)=ln\left(X_{0}\right)+\{ln\left(X_{max}\right)-ln\left(X_{0}\right)\}exp\left\{-exp\left[\frac{\mu_{max}e}{\{ln\left(X_{max}\right)-ln\left(X_{0}\right)\}}\left(\lambda -t\right)+1\right]\right\}$$

*X_t_*—the biomass concentration at time *t*, *X_0_*—the asymptotic biomass concentration at time zero, *X_max_*—the maximum biomass concentration, *μ_max_*—maximum specific growth rate, *λ*—lag time and *t*—time. 

The concentration of stressor required to inhibit the growth by 50% (*IC*_50_) was determined by Equation (2):

(2) $$\mu_{max}=\frac{{\left(\mu_{max}\right)}_{0}}{1+\left(c/IC_{50}\right)}\;\;\;\;\;$$

where *μ_max_* is the measured maximum specific growth rate at each stressor concentration (*c*), while (*μ_max_*)_0_ is the growth rate in the absence of stressor.

### 2.2. Protein Production and Purification

The *pden_3133* or *pden_3339* (ArsH; GI: 119376450), *pden_3136* (ArsR1; GI: 119376453) and *pden_3342* (ArsR2; GI: 119376652) gene cloning, expression and purification of the protein with an N-terminal six-histidine tag in *E. coli* BL21(DE3)pLysS (Thermo Fisher Scientific, Waltham, MA, USA) were carried out using a pET100/D-TOPO (Thermo Fisher Scientific, Waltham, MA, USA) plasmid as described previously [20]. The primers used were FP-3133 (5′-CACCATGATCCCCGACTTGCCGAACCTGT-3′), RP-3133 (5′-TCATGTCGCAGCCTTGAGGTTTACCCGTT-3′), FP-3136 (5′-CACCATGGAAGAGCA-ACACGCCCTTGTCGG-3′), RP-3136 (5′-TCAGGCCTCGCGGGTCAAAGTGGGGTCAC-AGGCA-3′), FP-3342 (5′-CACCATGGACGAGCAACGCGCCCTTGCCGG-3′) and RP-3342 (5′-TCAGCCATGCAGGGCATCCTTGCCGGGTT-3′). The correct sequence of the amplified genes inserted into a pET100/D plasmid was confirmed by sequencing.

### 2.3. Determination of Protein Concentration

Protein concentration was determined by Pierce BCA Protein Assay Kit (Thermo Fisher Scientific, Waltham, MA, USA) using bovine serum albumin as the standard. Protein eluted from a chromatographic column was monitored at 280 nm.

### 2.4. Protein Crystallization

Crystallization of the recombinant Pden_3133 protein (ArsH) with N-terminal hexahistidine tag was performed at 25 °C by the sitting-drop vapor-diffusion technique (Table S1). Needle-like crystals about 10 μm wide and up to 120 μm in length typically grew within 3 days. All crystals were briefly (less than 2 s) soaked in glycerol based cryoprotectant solution before being frozen in liquid nitrogen for storage.

We were unsuccessful in co-crystallizing Pden_3133 with FMN. Attempts to induce the FMN ligand via soaking resulted in collapse of the crystals.

### 2.5. X-ray Data Collection and Processing

X-ray data collection was performed at the synchrotron Soleil Proxima-1 beamline equipped with the Pilatus-6M detector at a wavelength of 0.97857 Å at 100 K using a 0.1° rotation per image. All data were reduced using MOSFLM [21] and scaled using SCALA, both as part of CCP4 package (v7.1, STFC Rutherford Appleton Laboratory, Didcot, UK) [22].

### 2.6. Structure Solution and Refinement

The initial model was obtained using the BALBES [23] automated pipeline. This model was then used for another cycle of molecular replacement using PHENIX [24] and the structure of *Shigella flexneri* ArsH (the Protein Data Bank (PDB) code: 2FZV) as a template. The refinement was performed in multiple cycles using both PHENIX and REFMAC [25]. The final refinement however had to be performed manually in COOT [26] as the data contained a significant deal of ambiguity in regards of sidechain conformation of significant number of residues. Crystal structure parameters are summarized in Table S2.

### 2.7. arsH Gene Transcript Analysis

Growth in the presence of inorganic arsenics, organoarsenicals or redox stressors was performed in 100 mL conical flasks in 15 mL of succinate aerobic mineral medium supplemented with one of a series of concentration of effectors. The flasks were incubated at 30 °C, and shaken at 225 rpm until the OD_600_ rose to a final value of 0.6. The cells from 1 mL were harvested by centrifugation at 11,000× *g* for 1 min, and then immediately lysed using TRI reagent (Top-Bio, Prague, Czech Republic). Total RNA was cleared of DNA contamination with a TURBO Ambion DNAfree kit (ThermoFisher Scientific, Waltham, MA, USA). Approximately 1.5 μg of purified RNA was reversely transcribed to cDNA with random hexamer primers (ThermoFisher Scientific, Waltham, MA, USA) and M-MLV reverse transcriptase (Top-Bio, Prague, Czech Republic). Relative level of ArsH mRNA in individual samples was estimated by quantitative PCR on a Light Cycler 480 (Roche, Basel, Switzerland) with the specific primers for the *arsH* genes FP-RT-3133 (5′-GTCGAGCTATTACGACCGCA-3′) and RP-RT-3133 (5′-CAGCCTTGAGGTTTACCCGT-3′) and normalized to mRNA levels of housekeeping genes for glyceraldehyde 3-phosphate dehydrogenase (pden_1060, forward primer—5′-TTTCCTCGGACTTCAACCAC-3′, reverse primer—5′-CTCGTTGTCATACCAGGTCAG-3′), sigma factor 54 (pden_2604, forward primer—5′-GGCTCAAGGATCACTACAAGG, reverse primer—5′-TCCTGCGTTTCCCATTCATC-3′) and beta subunit of DNA polymerase III (pden_0970, forward primer—5′-GTGCCTATCCCGATTACACG-3′, reverse primer—5′-TCTAAATCGAACTTCAGGGCG-3′). One reaction mixture contained 2 μL of cDNA equivalent to 5–6 ng of total RNA, 4 μL of RNase-free water (Top-Bio, Prague, Czech Republic), 2 μL (10 pmol) of forward and reverse primer and 10 μL of 2xSYBR Master Mix (Top-Bio, Prague, Czech Republic). After an initial polymerase activation step at 95 °C for 15 min, PCR amplification was performed as follows: 40 cycles of DNA denaturation and annealing at 95 °C and 60 °C for 20 s and extension at 72 °C for 30 s, with three replicates for each sample analyzed. In each experiment, the C_t_ (cycle threshold) values for the gene of interest were normalized to the C_t_ of three housekeeping genes and averaged. The relative mRNA expression was calculated as fold change 2^−ΔΔCt^ [27].

### 2.8. Enzyme Assays

The steady-state kinetics measurements were carried out at 30 °C in a final volume of 1 mL or 3 mL in 0.1 M sodium phosphate buffer (pH 7.0). The NADPH oxidation rate was calculated from the initial slope of the absorbance time decrease at 340 nm in an UltroSpec 2000 spectrophotometer (Cytiva, Marlborough, MA, USA) using a molar absorption coefficient of 6220 M^−1^ cm^−1^ [28]. The kinetic parameters were estimated by the Marquardt–Levenberg nonlinear fir algorithm included in the OriginLab Origin2021b software (v9.8.5.201, OriginLab Corporation, Northampton, MA, USA). Initial rate data for enzymatic reactions involving two varying substrates were fitted to Equations (3) and (4) describing sequential and ping-pong mechanisms, respectively [29].

(3) $$v=\frac{V\left[A\right]\left[B\right]}{K_{a}\left[B\right]+K_{b}\left[A\right]+\left[A\right]\left[B\right]+K_{ia}K_{b}}\;\;\;$$

(4) $$v=\frac{V\left[A\right]\left[B\right]}{K_{a}\left[B\right]+K_{b}\left[A\right]+\left[A\right]\left[B\right]}\;\;\;$$

In Equations (3) and (4), *v* and *V* are initial and maximum velocities, respectively, [*A*] and [*B*] are reactant concentrations, *K*_a_ and *K*_b_ are Michaelis constants (=*K*_M_) for A and B, respectively, and *K*_ia_ the dissociation constant for the first substrate to bind.

The molecular activity *k*_cat_ was determined by dividing *V* by the molar enzyme concentration using a molecular mass of recombinant ArsH of 30 kDa.

Reduced FMN was prepared in situ from FMN in an anaerobic cuvette flushed with ultrapure argon by titrating with freshly prepared sodium dithionite solution injected via a gas-tight Hamilton microsyringe. The backward oxidation reaction was followed at 450 nm using an absorption coefficient for FMN of ε_450 nm_ = 12.2 mM^−1^ cm^−1^ [30].

The phenylarsonous reaction product was identified and quantified using HPLC analysis by a modified protocol derived from Chen et al., 2015 [17]. Then, 9 nM ArsH was incubated at 30 °C in a final volume of 3 mL of 100 mM sodium phosphate buffer (pH 5.5) with 150 μM NADPH, 50 μM FMN and 159 μM PhAsO(OH)_2_. At the indicated times, 0.15 mL portion was filtered through 10-kDa cutoff Amicon Ultrafilter (EMD Millipore, Billerica, CA, USA ). The filtrate was loaded onto a 7 μm C18 reverse-phase column (250 × 4 mm, Watrex, Prague, Czech Republic) connected to a HPLC chromatograph (HP 1200 series, Agilent, Santa Clara, CA, USA) and eluted isocratically with a mobile phase consisting of 3 mM malonic acid, 5 mM tetrabutylammonium hydroxide, and 30% methanol (*v*/*v*), pH 5.6, with a flow rate of 1 mL min^−1^ at 30 °C. PhAsO was monitored at 236 nm. The calibration line was constructed from the peak heights of a series of PhAsO standards.

### 2.9. ArsR Molecular Mass Determination

The molecular mass of ArsR protein ArsR1 or ArsR2 was measured using an ÄKTA Pure fast protein liquid chromatography system (Cytiva, Marlborough, MA, USA). First, 500 μL of 40 μM ArsR1 or 37 μM ArsR2 protein in 10 mM MOPS/0.1 M NaCl/15% glycerol buffer (pH 7.5) was loaded onto a Superose 12 column (Cytiva, Marlborough, MA, USA) pre-equilibrated by the same buffer at a flow rate 0.5 mL min^−1^ and elution was monitored at 280 nm. The calibration was carried out with gel filtration standards ribonuclease A, chymotrypsinogen A, ovalbumin, and albumin in a concentration of 1 mg mL^−1^. Then, 100 μM sodium arsenite or 1 mM diamide was mixed with the proteins and incubated for 10 min at 30 °C to investigate how these compounds influence ArsR oligomeric state.

### 2.10. Electromobility Shift Assay

The oligonucleotides 5′-labeled with fluorescein amidite (FAM) used for electromobility shift assays and fluorescence anisotropy measurements were as follows: Pden1-F, 5′-FAM-CTC AAC ATA TCT TGA GAG GTT GAT TCG-3′; Pden1-R, 5′-CGA ATC AAC CTC TCA AGA TAT GTT GAG-3′; Pden2-F, 5′-FAM-ATC AAC ATA TCT TGA GAG GTT GAT TCG-3′; Pden2-R, 5′-CGA ATC AAC CTC TCA AGA TAT GTT GAT-3′. To generate double-stranded oligonucleotides, PDNA1 and PDNA2, an equal amount of the complementary single-stranded oligonucleotides was mixed, heated to 60 °C for 1 min, and annealed by gradually cooling down to ice temperature. Electromobility shift assays were performed in 30 µL of the binding reaction mixture containing 10 µM PDNA1 or PDNA2 and 10 µM ArsR1 or ArsR2 in a binding buffer (10 mM Tris-HCl, 50 mM NaCl, 0.5 mM EDTA, pH 8.0) and, where appropriate, 1 mM sodium arsenite or 1 mM diamide. The mixture was incubated at room temperature for 30 min. Then 6 µL of the loading buffer (0.25% bromophenol blue, 0.25% xylene cyanol, 30% (*v*/*v*) glycerol) was added. The reaction products were analyzed by electrophoresis in a 3% agarose gel supplemented by 0.5 µg mL^−1^ of ethidium bromide in ×1 Tris/acetate/EDTA buffer at constant 100 V for 2 h and at 7 °C. After the electrophoresis, bands containing DNA were visualized under UV light in a Fusion FX transilluminator (Vilber, Collégien, France) and the image was processed by an Evolution-Capt Edge software (Vilber, Collégien, France). The marker was 100 bp DNA ladder purchased from Promega (Promega, Madison, WI, USA). 

### 2.11. Fluorescence Anisotropy Measurement

The fluorescence anisotropy of FAM labelled oligonucleotides PDNA1 or PDNA2 was measured in a FluoroMax-4 spectrofluorometer (Horiba Scientific, Kyoto, Japan) equipped with polarizers operated by FluorEssence software version 3.5 (Horiba Scientific, Kyoto, Japan) and calculated from the intensities at four mutually perpendicular polarizer settings. Excitation was at 493 nm, and the emission was recorded at 517 nm. A fixed amount of 5′-FAM-oligonucleotides was titrated with aliquots of ArsR proteins of known concentrations in 10 mM MOPS/0.1 M NaCl/15% glycerol (pH 7.5) at 30 °C. The dissociation constant *K*_d_ were calculated by nonlinear regression analysis according to a 1:1 binding model [31] using Equation (5).

(5) $$A=A_{{\mathrm{DNA}}_{F}}+\left(A_{P-\mathrm{DNA}}-A_{{\mathrm{DNA}}_{F}}\right)\left\{\frac{K{\left[DNA\right]}_{T}+K{\left[P\right]}_{T}+1-\sqrt{{\left(K{\left[DNA\right]}_{T}+K{\left[P\right]}_{T}+1\right)}^{2}-4K^{2}{\left[DNA\right]}_{T}{\left[P\right]}_{T}\;}}{2K{\left[DNA\right]}_{T}}\right\}$$

*A*—the measured value of anisotropy, *A*_DNAF_—a specific value of anisotropy associated with free DNA, *A*_P-DNA_—a specific value of anisotropy associated with the complex of DNA and the protein, [*DNA*]_T_—the total concentration of DNA, [*P*]_T_—the total concentration of the protein and *K*—the association constant. 

## 3. Results

### 3.1. arsH Genes in the Genus Paracoccus

7 out of the 13 genomes of the genus *Paracoccus* available on the KEGG database contain one copy of a putative ars operon comprising the *arsH* gene and two of them (the genomes of *P. denitrificans* and *P. zhejiangensis*) contain two copies of such an operon. The *P. denitrificans* ars operon-like gene clusters are of the *arsRCBH* type, where *arsR* codes for a putative repressor, *arsC* for an As(V) reductase, *arsB* for an As(III) efflux pump and *arsH* for a flavin reductase. These clusters (genes 3131 to 3136 and 3337 to 3342) were denoted as *ars1* and *ars2*. The predicted protein products of *arsH1* and *arsH2* genes (pden_3133 and 3339) are composed of 235 amino acids and display 100% sequence identity with each other. There is also significant sequence homology with the most intensively studied ArsH proteins from other organisms (Figure S1).

### 3.2. ArsH Structure

*P. denitrificans* ArsH was produced in *E. coli* as an N-terminal 6xHis tag fusion, purified by affinity chromatography and crystallized by the sitting-drop vapor-diffusion technique (Figure S2). The crystal structure was determined at 2.6 Å resolution using molecular replacement with the ArsH model of *Shigella flexneri* [14] and deposited in the Protein Data Bank (PDB) under the identifier code 7PLE. 

The asymmetric unit of the crystal structure of *P. denitrificans* ArsH contains four monomers, named A, B, C and D, tightly packed together into a tetramer (Figure S3). A dimer is the usual form of flavodoxins and a dimer of dimers is assumed to be a biological assembly of *S. flexneri* ArsH [14]. The same holds for *P. denitrificans* ArsH. Crystal packing analysis using PISA [32] gave the following values of the interface area between monomers: D:C 2222.9 Å^2^, B:A 2158.1 Å^2^, D:B 1255.0 Å^2^, C:A 1244.0 Å^2^, C:B 232.6 Å^2^ and D:A 230.2 Å^2^. The pairs A, B and C, D thus can form structurally cohesive dimers and the dimer–dimer interaction is dictated by the combined A-C and B-D interfaces.

Each individual monomer comprises a five-stranded parallel β-sheet surrounded by five α-helices and a short helical loop rich in hydrogen bonds. The structure of the monomer can be divided into two sections, the main body, where the β-sheet and its flaking α-helices are located and where the active site is predicted to be, and the C and N terminals that form a structure binding to the neighbor monomer.

**Figure 1:**
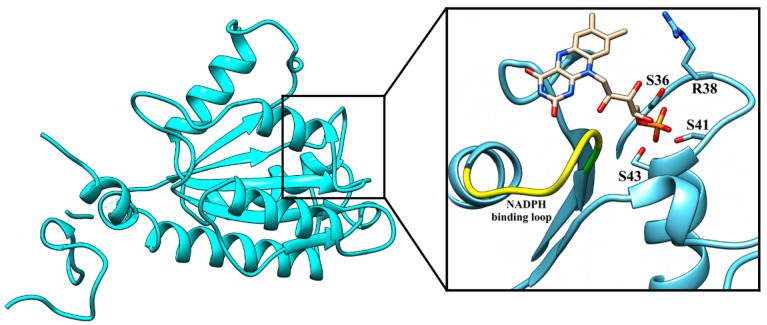
Overall structure of ArsH from *P. denitrificans* and the putative active site domain. The image was generated with Chimera 1.13.1 on the basis of the ArsH crystal structure (7PLE). Superposition of FMN from FerB (white) and ArsH in its an active site (zoomed in the square). The 3D coordinate data of FMN were taken from the PDB file 3U7R. The amino acids that probably interact with the phosphate group of the coenzyme and the putative NADPH binding loop are shown.

The mode of substrate binding can be tentatively predicted by similarity to other enzymes. The sequence GSLRERSYS located in the β1-α1 loop and N-terminus of α1 (the underlined letters represent the residues conserved in ArsH proteins) matches the consensus site GSXRXXS for binding of the phosphate group of FMN [33]. In Figure 1, FMN binding was modeled by structural superposition with the flavoenzyme FerB from the same bacterium (PDB ID: 3U7R; [2]). The β4-α4 loop protruding over the presumed FMN binding site carries residues that are highly conserved among the ArsH proteins and partly conform to the consensus sequence [VICL]-X-[IVC]-X-G-G-X2-[VIL]-[YFA]-X2-[AFMCLV]-[LMIVF] derived from the proteins known to specifically bind NADP^+^ [34].

### 3.3. Enzymatic Activities of ArsH

**Figure 2:**
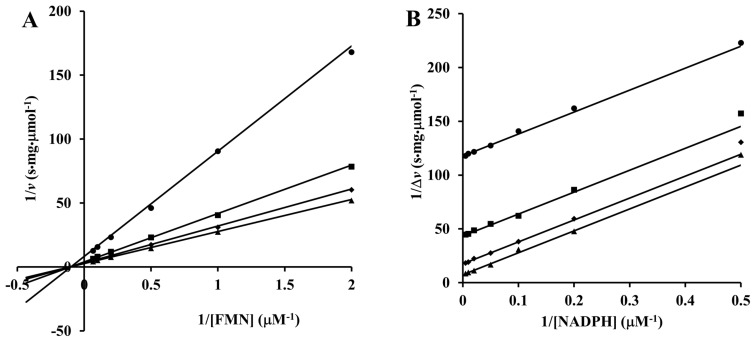
Double reciprocal plots from initial velocity data of the ArsH reactions. Measurements were performed at 30 °C in 0.1 M sodium phosphate buffer (pH 7.0). The reaction was started by the addition of ArsH, and the decrease in absorbance at 340 nm was recorded. Plot (**A**), NADPH:FMN oxidoreductase. ArsH concentration, 205 nM. The fixed concentrations of NADPH are 5 (circles), 20 (squares), 50 (rhombs) and 150 μM (triangles). Plot (**B**), NADPH:PhAsO(OH)_2_ oxidoreductase. ArsH concentration, 1.4 μM. FMN concentration, 50 μM. The fixed concentrations of PhAsO(OH)_2_ are 10 (squares), 30 (circles), 100 (rhombs) and 1000 μM (triangles).

The catalytic properties of ArsH were determined from the steady state kinetic measurements. Figure 2A shows the results of bisubstrate kinetic analysis, presented as a double reciprocal plot. A family of straight lines of different slopes and intercepts with one common intersection point is indicative of a sequential type mechanism which includes the formation of a ternary complex of FMN and NADPH with ArsH prior to the catalytic event. The kinetic parameters obtained from a global fit of all data to a sequential kinetic model (Equation (3)) are *k*_cat_ = (12.6 ± 0.4) s^−1^, *K*_M_^FMN^ = (9.3 ± 0.7) μM and *K*_M_^NADPH^ = (12 ± 2) μM.

As discussed in the previous paper [9], enzymes such as ArsH can secondarily reduce various electron acceptors via the reduced FMN formed. If the reaction takes place on the enzyme surface, the acceptor behaves as a regular substrate, that is, its concentration influences the initial rate of NAD(P)H consumption in a saturable fashion. On the contrary, the reaction of acceptor with the reduced FMN already released from the enzyme is purely chemical and does not affect the initial rate of the preceding enzymatic step. In contrast to results seen previously for Pden_5119, an ArsH homolog that functions as an NADH oxidase [9], the rate of NADPH oxidation by ArsH turned out to be oxygen independent (Figure S4). An explanation may be that the reduced flavin formed in the active site is oxidized by oxygen only after its release from the enzyme.

In case of *P. denitrificans* ArsH, the NADPH oxidation rate accelerated upon addition of phenylarsonic acid PhAsO(OH)_2_. The extra initial velocity Δ*v*, obtained by taking the difference of the initial velocities in the presence of phenylarsonic acid and in its absence, was subjected to a bisubstrate kinetic analysis (Figure 2B). The double reciprocal plot of Δ*v* as a function of variable concentrations of NADPH at different fixed concentrations of phenylarsonic acid and at a constant concentration of FMN yielded parallel lines typical of ping-pong reaction mechanisms. The apparent values *k*_cat_, *K*_M_^NADPH^ and *K*_M_^PhAsO(OH)2^, as calculated by nonlinear regression (Equation (4)), are (4.9 ± 0.1) s^−1^, (32 ± 1) μM and (176.7 ± 4.3) μM, respectively.

**Figure 3:**
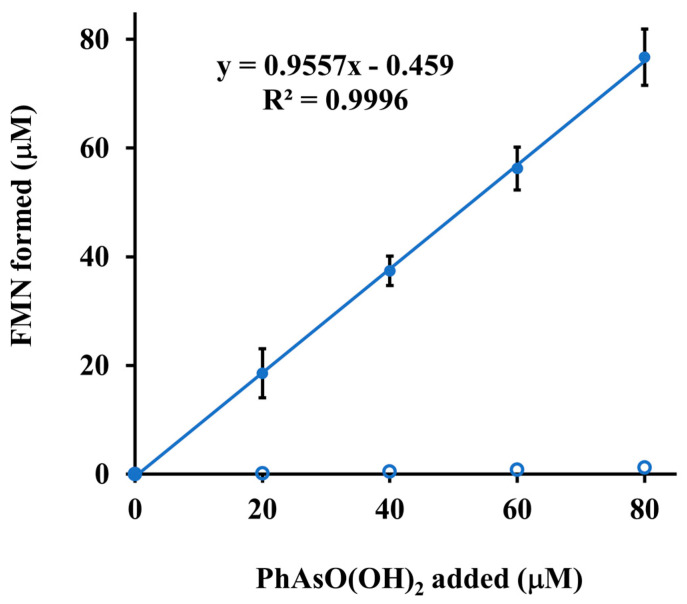
ArsH-catalyzed oxidation of reduced FMN by phenylarsonic acid. A sealed 1 cm spectrophotometric cuvette contained 1 mL of argon-purged 0.1 M sodium phosphate buffer, pH 7.0, with 100 μM FMN. 205 nM ArsH was either present (solid circles) or absent (open circles). After reduction of FMN with dithionite, measurement was performed by adding successive 20 μM portions of phenylarsonic acid. The resulting absorbance at 450 nm was read after attaining a constant level (within about 5 min). All A_450_ readings were corrected by subtracting the initial absorbance and converted to concentrations using an absorbance coefficient of 12,200 M^−1^ cm^−1^. The value of the slope of the straight line fitted to the data points indicates that the stoichiometric ratio of FMN produced to phenylarsonic acid added is 1:1. Each data point is mean of triplicate determinations ± SD.

**Figure 4:**
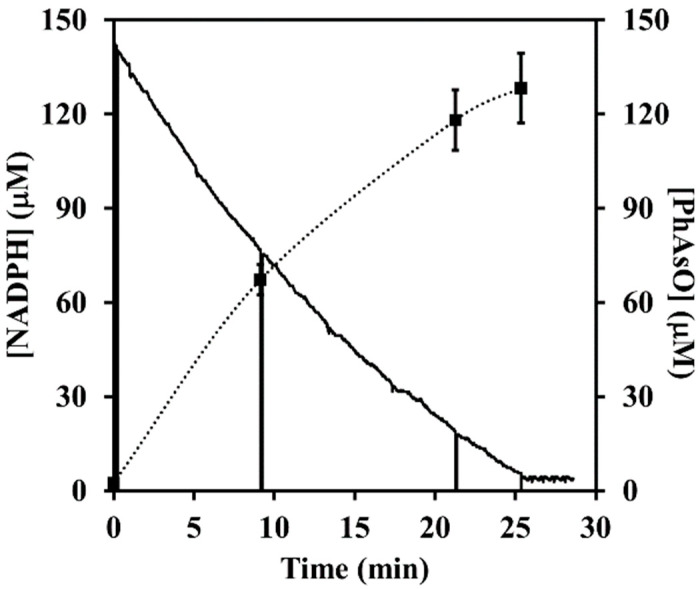
Time courses of NADPH consumption and arsenous product accumulation. The reaction mixture initially contained 142 μM NADPH, 50 μM FMN, 159 μM PhAsO(OH)_2_ and 9 nM ArsH in 0.1 M sodium phosphate buffer (pH 7.0, 30 °C). Oxidation of NADH was continuously monitored at 340 nm (solid line) while samples were taken for HPLC analysis of the phenylarsonous acid levels (points lying on the dotted line). Vertical bars denote standard deviations from triplicate samples.

The observed ping-pong kinetics suggests that ArsH is competent for catalysis of not only FMN reduction but also its re-oxidation. The participation of an oxidative half-reaction in the catalytic cycle of ArsH was directly demonstrated by spectrophotometric monitoring of the conversion of FMNH_2_ to FMN elicited under anaerobic conditions by the arsenic substrate (Figure 3). The enzymatic nature of this reaction was clearly established by the fact that there was no detectable production of FMN in the absence of ArsH. The observed 1:1 stoichiometry between FMN produced and phenylarsonic acid added is consistent with a two-electron reduction to give phenylarsonous acid PhAs(OH)_2_, the hydrated form of phenylarsine oxide PhAsO. Using authentic phenylarsine oxide as a standard, we showed by HPLC analysis of aliquots taken at various times from the complete reaction mixture containing NADPH, phenylarsonic acid, FMN and ArsH that the postulated reaction product was indeed formed along with NADPH consumption (Figure 4).

For comparison, we also examined in similar experiments the effects of addition of 100 μM PhAsO instead of PhAsO(OH)_2_. There was no change in ArsH-catalyzed NADPH oxidation rate in the presence of FMN, irrespective of whether oxygen was absent or present. We also did not observe any significant anaerobic FMN reduction by PhAsO, either enzymatic or non-enzymatic.

### 3.4. arsH Gene Expression Elicited by Arsenicals

**Figure 5:**
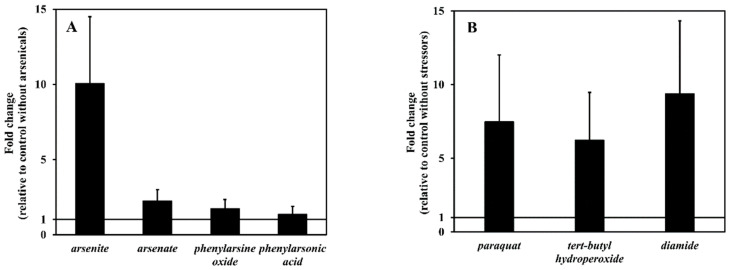
Transcriptional level analysis of the *arsH* gene. Panel (**A**), the effect of various arsenicals and phenylarsenicals; panel (**B**), the effect of various oxidative stressors. Bars represent the mean ± SD for three measurements. Relative mRNA level of the *arsH* gene were normalized to three house-keeping genes and quantified relative to the wild-type strain without any effectors.

To study the response of *arsH* to the representative inorganic and organic arsenic compounds, growth inhibition experiments were first conducted. The IC_50_ values obtained (Equation (2)) were 5.0 ± 0.5 µM, 97 ± 5 mM, 0.41 ± 0.03 µM and 4.4 ± 0.1 mM for arsenite, arsenate, phenylarsine oxide, and phenylarsonic acid, respectively. Based on these results, a subtoxic concentration was calculated for each compound, defined as the concentration that causes a decrease in the maximum specific growth rate by 10%. Exposure of the exponentially growing cells to the subtoxic concentrations of arsenicals for 5–6 h until an OD_600_ of 0.6 was reached, resulted in alterations in the transcript level of *arsH* presented in Figure 5A. It can be seen that arsenite was highly effective in elevating *arsH* mRNA whereas the other compounds showed a relatively low efficacy. 

### 3.5. arsH Gene Expression Elicited by Redox Stressors

In a previous work we observed that generation of superoxide through redox cycling was associated with increased accumulation of the Pden_3133 protein [8]. This observation is further corroborated by measuring *arsH* transcript in cells after challenge with oxidative stress inductors paraquat, tert-butyl hydroperoxide and diamide. Figure 5B shows that all these stressors exerted a significant enhancing effect comparable to that of arsenite.

### 3.6. Synergistic Growth Inhibitory Effects

**Figure 6:**
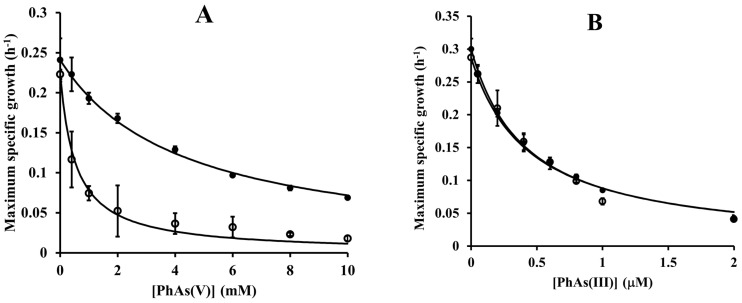
The effect of arsenite on the cell sensitivity to phenylarsenicals. Various concentration of phenylarsonic acid (panel (**A**)) or phenylarsine oxide (panel (**B**)) were incubated with *P. denitrificans* 1222 in the absence (closed circles) or presence (open circles) of 0.5 mM arsenite. The maximum specific growth values represent means ± SD of three biological replicates. IC_50_ values for phenylarsonic acid were 4.26 ± 0.11 mM without arsenite and 0.54 ± 0.06 mM with arsenite, and for phenylarsine oxide were 0.42 ± 0.02 mM without arsenite and 0.44 ± 0.03 mM with arsenite.

We next examined how increased intracellular level of ArsH would affect the toxicity of organic arsenicals. We therefore designed an additional growth inhibition experiment in which the *ars* operon was first derepressed by arsenite and only then the effect of organoarsenicals on growth was evaluated. We reasoned that if ArsH catalyzes the reduction of As(V) to the more toxic As(III), the inhibition capacity of As(V) derivatives should increase. On the other hand, if ArsH catalyzes the oxidation of As(III) to As(V), the inhibition capacity of As(III) should be weakened. From the results in Figure 6, it can be inferred that a sub-toxic concentration of arsenite synergistically reduced the IC_50_ value for phenylarsonic acid by 7.9-fold, whereas the IC_50_ value for phenylarsine oxide did not vary significantly (*p* > 0.05). Thus, these results indicate an As-reducing rather than an As-oxidizing activity of the ArsH protein under in vivo conditions.

### 3.7. Effect of Heterologously Expressed arsH Gene

**Figure 7:**
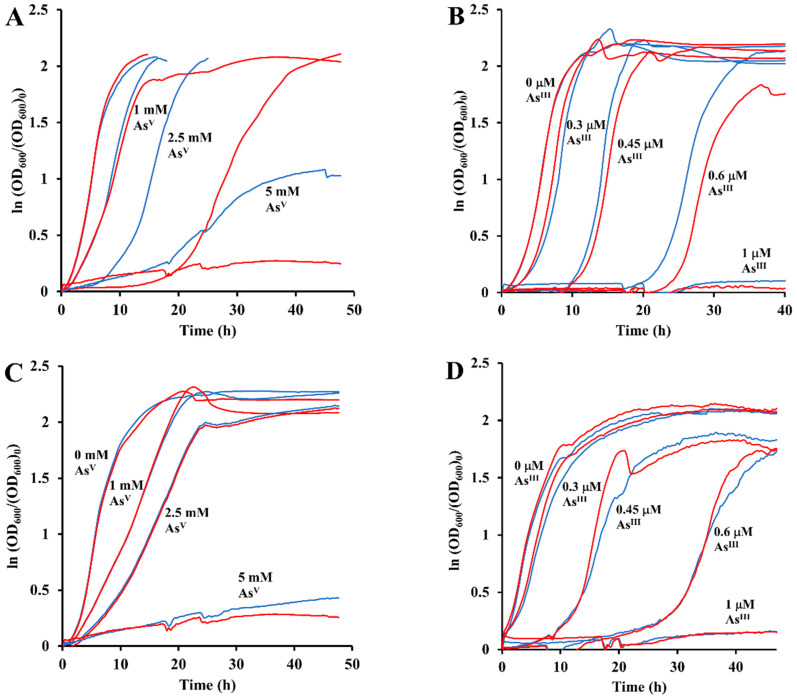
The averaged (*n* = 3) growth curves of *E. coli* BL21(DE3)pLys harboring the *arsH* gene (panels (**A**,**B**)) and the negative control lacking it (panels (**C**,**D**)) cultured in M9 medium with glucose with the addition of the indicated concentration of PhAsO(OH)_2_ (panels (**A**,**C**)) or PhAsO (panels (**B**,**D**)). Blue curves and red curves have been obtained in the absence and in the presence of 1 mM IPTG, respectively.

In order to further validate the above hypothesis, we performed heterologous expression experiments in *E. coli*, the *ars* operon of which does not encode an ArsH protein. *E. coli* BL21(DE3)pLys cells were transformed with either the parental pET plasmid having no insert or the pET plasmid carrying the *pden_3133* gene and cultured on microplates without or with 1 mM IPTG in media containing various concentrations of phenylarsonic acid. Averaged (*n* = 3) time courses of turbidity changes are depicted in Figure 7. Similarly, as shown for *P. denitrificans*, phenylarsonic acid inhibited growth of *E. coli* at the millimolar range, while three orders lower concentrations of its reduced trivalent form were already effective. The growth curves at zero arsenic concentration had mutually similar profiles, which indicates that neither IPTG alone nor the ArsH protein accumulated in cells induced by IPTG suppressed cell growth. ArsH-expressing transformant (grown with IPTG present) exhibited a significantly higher sensitivity to 2.5 and 5 mM concentrations of the pentavalent organoarsenic compared to the controls without IPTG or the *pden_3133* gene. This was manifested by prolonged lag phase and decreased growth rate. No differences in maximum specific growth rates were observed in experiments with phenylarsine oxide, suggesting that the toxicity of this compound was not significantly influenced by ArsH. Altogether, these results add further evidence in favor of ArsH-mediated reductive activation in organoarsenic toxicity.

### 3.8. ArsR Repressor Functioning

Regulation of the *ars* operon is known to be mediated by the ArsR protein, a repressor which binds to the *ars* operator and interferes with transcriptional initiation. Arsenic salts of the trivalent oxidation state interact with the homodimeric ArsR protein to release it from the *ars* operator site [35]. The ArsR proteins ArsR1 and ArsR2, encoded by the *pden_3136* and *pden*_*3342* genes, are similar in residue length (119 and 120) and highly homologous in sequence (identities = 103/114 (92%), E-value = 1 × 10^−76^). A bioinformatic search using Phyre 2 identified similarity with the crystal structure of *Acidithiobacillus ferrooxidans* ArsR (PDB ID: 6J05; [36]) which then served as a template for structural modeling. The structural model (Figure S5) retains much of the three-dimensional architecture of the template, including the position of helices 1 and 5, which are involved in dimerization. The C-terminus could not be modeled using the template. Nevertheless, multiple alignment of amino acid sequences (Figure S5) demonstrated in both *P. denitrificans* ArsR proteins the presence of conserved cysteine residues that bind As(III) in the *A. ferrooxidans* ArsR (Figure S5). The genes *pden_3136* and *pden_3342* encoding ArsR1 and ArsR2 were successfully expressed in *E. coli* as pET100 fusion proteins tagged with hexahistidine at their N-termini. 

Previous studies have defined the core consensus motif of binding sites of ArsR from other bacteria as ATCAAN_6_TTGAT [37]. Inspection of the conserved DNA region, (C/A)TCAACATATCTTGAGAGGTTGATTCG, lying upstream of the *ars* operons just before the translational start ATG codon, revealed the presence of two putative operator sites, (C/A)TCAACATATCTTGAG and ATCTTGAGAGGTTGAT, that overlap one another by 8 bp. 

**Figure 8:**
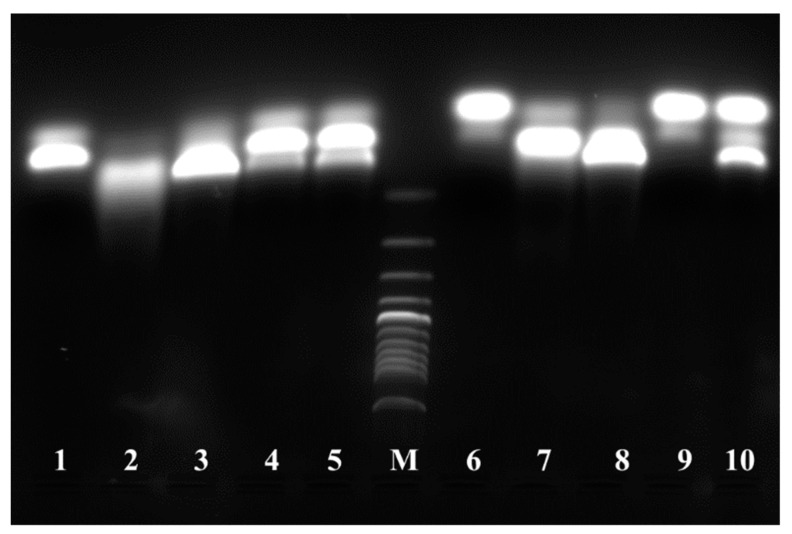
Electrophoretic shift assay with a 27-bp *arsR* promoter DNA fragments with ArsR proteins. FAM-labeled DNA fragments were incubated with or without purified ArsR proteins for 30 min at room temperature. The mixtures were electrophoresed on 3% agarose gels at 7 °C with ethidium bromide and visualized by UV-transilluminator. When either 10 μM PDNA1 (lane 1) or PDNA2 (lane 6) was incubated with the recombinant protein 10 μM ArsR1 (lane 2 and 3) or ArsR2 (lane 7 and 8), retardation due to DNA-protein complex formation was observed. The retardation was almost suppressed in the presence of 1 mM sodium arsenite (lanes 4 and 9) or 1 mM diamide (lanes 5 and 10) for both complexes. M means the marker and it was 100 bp DNA ladder from Promega.

**Figure 9:**
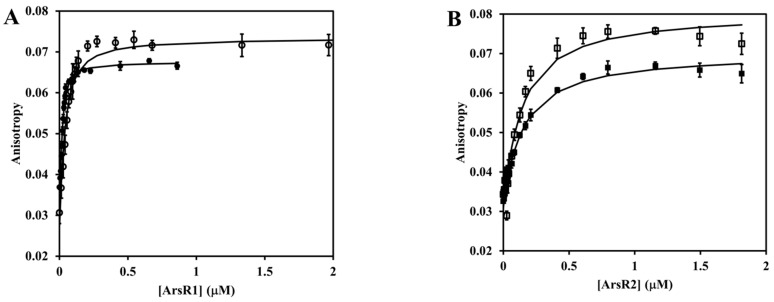
ArsR binding to DNA labelled by fluorescence probe. Titration was conducted in 10 mM MOPS/0.1 M NaCl/15% glycerol, pH 7.0 at 30 °C. Panel (**A**), 25 nM concentration of PDNA1 (•) or PDNA2 (◦) was titrated with the indicated concentrations of the recombinant protein ArsR1 protein. Panel (**B**), 25 nM concentration of PDNA1 (▫) or PDNA2 (▪) was titrated with the indicated concentrations of the recombinant ArsR2 protein. Solid lines represent the best fit of the data to Equation (5). The fitted parameters are presented in the main text. Each data point is a mean of ten determinations ± SD.

To test their functionality, 27-bp double-stranded oligonucleotide probes PDNA1 and PDNA2 spanning these sites were prepared and the binding of ArsR proteins was evaluated by using two independent methods, i.e., fluorescence gel retardation and fluorescence anisotropy. The data in Figure 8 show that incubation of either oligonucleotide probe with ArsR proteins resulted in a significant downshift of mobility, which can be ascribed to a complex formation. This did not occur when the proteins were pre-exposed to 1 mM of diamide or 0.1 mM of sodium arsenite, suggesting that both treatments were disruptive to protein-DNA binding. Quantitative insight into the binding interactions was provided by the fluorescence anisotropy profiles (Figure 9). These measurements take advantage of the fact that the rotational motion of the fluorescein-labeled oligonucleotide is restricted due to its attachment to the protein, which manifests itself as an increase in anisotropy. Curve-fitting to a 1:1 binding model (Equation (5)) gave the following dissociation constants (nM): ArsR1-PDNA1, 9 ± 1; ArsR1-PDNA2, 26 ± 4; ArsR2-PDNA1, 130 ± 20; ArsR2-PDNA2, 140 ± 10. It is therefore evident that both ArsR proteins recognize the promoter of not only its own but also the other ars operon. ArsR1 is a stronger binder and exhibits higher discrimination capability for promoter recognition than ArsR2. The addition of 10 mM arsenite or 1 mM diamide to the preformed ArsR-DNA complex caused the anisotropy to quickly drop down with a half-time of about 200 s in line with what is expected for a dissociation of the complex. 

**Figure 10:**
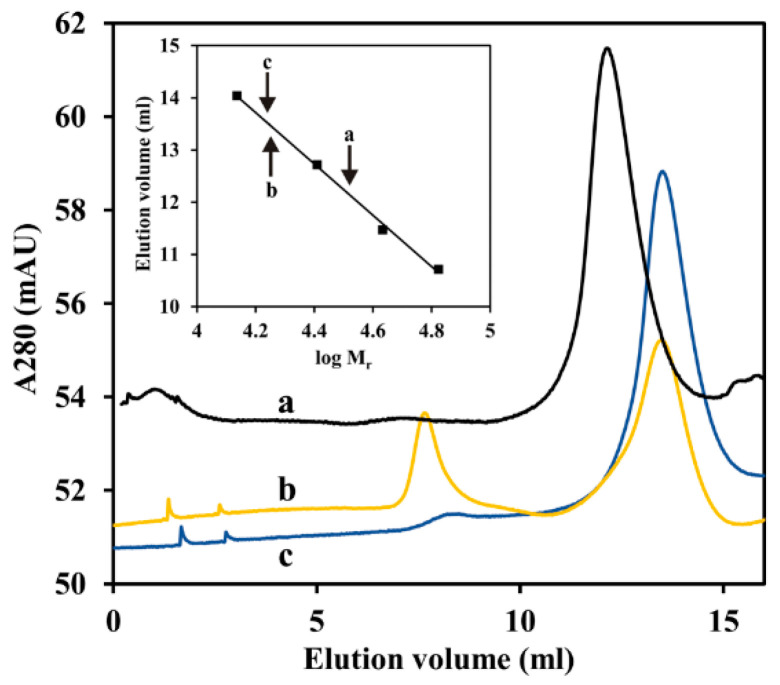
Determination of the molecular mass of diamide or arsenite treated ArsR1. First, 0.5 mL of sample (1.0 mg mL^−1^ of proteins) was loaded onto a Superose 12 10/300 GL (GE Healthcare) analytical and preparative column equilibrated by 10 mM MOPS/0.1 M NaCl/15% glycerol (pH 7.5). The flow rate after sample application was 0.5 mL min^−1^. Marker proteins (solid squares in inset graph) were albumin (66.7 kDa), ovalbumin (43 kDa), chymotrypsinogen A (25.7 kDa) and ribonuclease A (13.7 kDa). Line a, ArsR1 alone; line b, ArsR1 in the presence of 1 mM diamide; line c, ArsR1 in the presence of 0.1 mM arsenite. Molecular masses calculated from the calibration curve were 33.1 kDa (a), 16.8 kDa (b) and 16.6 kDa (c). The theoretical molecular mass of the His-tagged monomer is 16,744 Da.

ArsR repressor proteins from other sources are reported to exist in two forms, dimeric and monomeric, with the dimer being the only repressive form [38]. To determine the oligomeric status of *P. denitrificans* ArsR, gel permeation HPLC was carried out (Figure 10). The ‘native’ recombinant ArsR protein eluted as a single peak at a position corresponding to ∼32 kDa, which is approximately double the predicted molecular mass of an ArsR monomer (16.7 kDa). In contrast, arsenite or diamide-treated ArsR eluted at the monomer size, although in case of diamide a minor high molecular mass fraction was also detectable. These findings support the view that the observed loss of DNA binding ability is in both cases caused by monomerization.

## 4. Discussion

NADPH-dependent flavin reduction proceeding through a sequential ternary-complex mechanism and typical structural features indicate that the *P. denitrificans* ArsH does not fundamentally differ from the other ArsH proteins known so far. Our current demonstration that this type of enzyme also acts as an organoarsenical reductase is therefore of wider importance in relation to arsenic metabolism and toxicity in bacteria. The half-cell potential, E_1/2_, of phenylarsonic acid was estimated as 441 mV at 23 °C and pH 3.0 [39]. From the known pKa values of 3.57 and 8.74 [40] it can be calculated that the standard redox potential at neutral pH is about 100 mV, which confirms the thermodynamic feasibility of the reduction by biological reducing agents such as NAD(P)H or glutathione with redox potentials of −324 mV and −263 mV, respectively [41]. Knowles [42] described the reduction of phenylarsonic acid and its derivatives by NADPH catalyzed by glutathione reductase. In the course of the reaction, the enzyme became inactivated by the accumulating arsenous product which covalently modified essential vicinal thiol groups at the active site. ArsH appears to be less sensitive towards product inhibition possibly because its two cysteine residues are well separated in the amino acid sequence and located spatially far away from the flavin cofactor-binding region. 

Our results contrast with those previously reported for ArsH from *Pseudomonas putida* [17]. In that study, ArsH was found to catalyze oxidation of trivalent organoarsenicals in the presence of NADPH, FMN and oxygen thus acting as a monooxygenase. Based on this finding and on the observed increased sensitivity of *arsH* gene deletion strains to organoarsenic(III) compounds, the authors hypothesized that the main biological function of ArsH lies in converting the trivalent forms of the organoarsenicals to the relatively less toxic pentavalent species. The exact reason for the difference in behavior of the ArsH ortholog from *P. denitrificans* is uncertain. It may be that the active site of *P. putida* ArsH is better suited for the formation and stabilization of C4a-hydroperoxy-FMN, the key intermediate in flavin monooxygenase chemistry [43], than is the active site of *P. denitrificans* ArsH. Alternatively, involvement of FMNH·, the semiquinone form of FMN, may be considered. FMNH·/FMNH_2_ redox couple has higher reduction potential than FMN/FMNH_2_ couple (−101 mV vs. −207 mV; [44]), which allows FMNH· to oxidize inorganic As(III) [45]. It is, therefore, conceivable that under some assay conditions free flavin may cycle back and forth between semiquinone and hydroquinone and shuttle electrons from As(III) to oxygen. If this is the case, ArsH would only provide reduced flavin needed to sustain the subsequent non-enzymatic electron transfer cycle. This possibility is consistent with our observation that the rate of oxidation of NADPH by ArsH under aerobic conditions was unaffected by PhAsO. 

There are three main arguments against accepting *P. denitrificans* ArsH as a detoxifying enzyme for trivalent organoarsenicals. First, ArsH actively catalyzes the reaction in the reductive direction (i.e., leading to the formation of the more toxic product). This possibility was seemingly not explored before for the *P. putida* enzyme. Second, neither phenylarsine oxide nor phenylarsonic acid upregulates *arsH* gene expression when added in subtoxic levels to growing culture of *P. denitrificans*. Third, derepression of *arsH* by arsenite significantly enhances the toxic effect of phenylarsonic acid on bacterial growth. This effect is not restricted to *P. denitrificans*, as it was also shown in *E. coli*. On the other hand, the toxicity of phenylarsine oxide does not decrease, which would be expected if ArsH had a protective potency. From this it appears that ArsH can be preferably characterized as a metabolic activator generating toxic reactive organoarsenic(III) species. The ArsH-catalyzed As(V)-to-As(III) conversion looks like an undesirable side process rather than a metabolically useful transformation, and hence, the true metabolic function of ArsH may be different.

Evidence is emerging for the importance of ArsH in oxidative stress defense. According to the results presented in this study, strong elevations in the *arsH* transcript level occur following exposure of *P. denitrificans* cells to redox stressors (Figure 5). In work with *P. putida* it was shown that the *arsH* genes strengthened the cells’ tolerance to both inorganic As(V) and As(III) and quenched the high intracellular levels of reactive oxygen species resulting from treatment of cells with arsenic oxyanions [18]. In this context, a possibility deserving consideration is that derepression of *ars* operons, and hence, the synthesis of ArsH does not necessarily need the presence of arsenic compounds, but can also take place under general oxidative stress conditions. This idea receives strong support from our experiments showing that both arsenite and the specific thiol oxidant diamide exert similar effects on the ability of the transcriptional repressor ArsR to bind to target DNA. The As (III)-responsive ArsR of *Acidithiobacillus ferrooxidans* senses arsenite through binding to the thiol groups of cysteines 95, 96 and 102. All three residues are retained in *P. dentrificans* ArsR proteins as Cys92, Cys93 and Cys100 (Figure S5), so that the interaction with As(III) may proceed in a similar way. The mentioned residues with two additional ones (Cys109 and Cys111) may also be involved in the redox regulation. Redox sensing by ArsR family proteins via their thiol groups has precedents in the literature, for instance the transcriptional repressors of metallothionein [46], thioredoxin [47], nitroreductase [48] and hydrogen sulfide detoxification proteins [49]. References [33,34,36,50,51] are cited in the Supplementary Materials.

## 5. Conclusions

To summarize, we have examined the role of the ArsH protein and shown that at least in *P. denitrificans* it acts as an organoarsenic reductase. Moreover, we have gained new insights into the regulation of *ars* operons by demonstrating a redox modulation of the activity of the transcriptional repressor ArsR. Further work is needed to elucidate mechanistic details regarding these proteins.

## Data Availability

Data is contained within the article or Supplementary Material.

## Supplementary Materials

The following supporting information can be downloaded at: https://www.mdpi.com/article/10.3390/antiox11050902/s1; Table S1: Crystallization conditions; Table S2: Data collection and refinement statistics; Figure S1: Multiple sequence alignment of *P. denitrificans* ArsH with homologous proteins; Figure S2: Crystals of *P. denitrificans* ArsH under VIS and UV illumination; Figure S3: The crystal structure of *P. denitrificans* ArsH tetramer and monomer; Figure S4: Dependence of the rate of NADPH oxidation, as catalyzed by ArsH on oxygen concentration; Figure S5: Sequence alignment and a model structure of the PdenArsR.
