# Supplementary material for: The ArsH Protein Product of the Paracoccus denitrificans ars Operon Has an Activity of Organoarsenic Reductase and Is Regulated by a Redox-Responsive Repressor
Source: Antioxidants (Basel). 2022 May 3;11(5):902. doi: 10.3390/antiox11050902 (PMC9137774; doi:10.3390/antiox11050902)
Supplement: Supplementary file 1 [file antioxidants-11-00902-s001.zip › antioxidants-1662101-supplementary.pdf]

Supplementary Materials

The ArsH protein of *Paracoccus denitrificans* has the activity of organoarsenic reductase and is regulated by a redox-responsive repressor

Vojtěch Sedláček, Martin Kryl and Igor Kučera

Department of Biochemistry, Faculty of Science, Masaryk University, Kotlářská 2, 611 37 Brno

Czech Republic

Supplementary Tables

Table S1. Crystallization conditions

Method	Vapor diffusion in sitting drop configuration
Plate type	Invoplate SD 2 (Molecular Dimensions)
Temperature (K)	298.15
Protein concentration	140 M
Buffer composition of protein solution	50 mM trisodium phosphate, 300 mM sodium chloride, 300 mM imidazole, pH 8.0
Composition of reservoir solution	4.77 M sodium acetate, 3% w/v 6-aminohexanoic acid, 80 mM HEPES pH 6.9
Volume and ratio of drop	200 nL, 1:1
Volume of reservoir	3500 nL

Table S2. Data collection and refinement statistics

Space group	$P3_2\,2\,1$
Unit-cell Parameters (Å)	*a* = 112.95
	b = 112.95
	c = 204.41
Wavelength (Å)	0.97857
Resolution range (Å)	97.81–2.60 (2.6930–2.6001)
Completeness (%)	99.9 (100)
Redundancy	2
σ cutoff	F > 1.440σ(F)
No. Reflections, working set	45145 (4456)
No. of reflections, test set	2011 (195)
Final R_{cryst}	0.2794 (0.4095)
Final R_{free}	0.2977 (0.4326)
Cruickshank DPI	0.438
No. non-H atoms	
Protein	6789
Water	185
Total	6974
RMS deviations	
Bonds (Å)	0.0237
Angles (°)	1.067
Average B factors (Å^2)	
Protein	26.6
Water	47.3
Ramachandran plot	
Most favoured (%)	96.82
Allowed (%)	2.47

Supplementary Figures

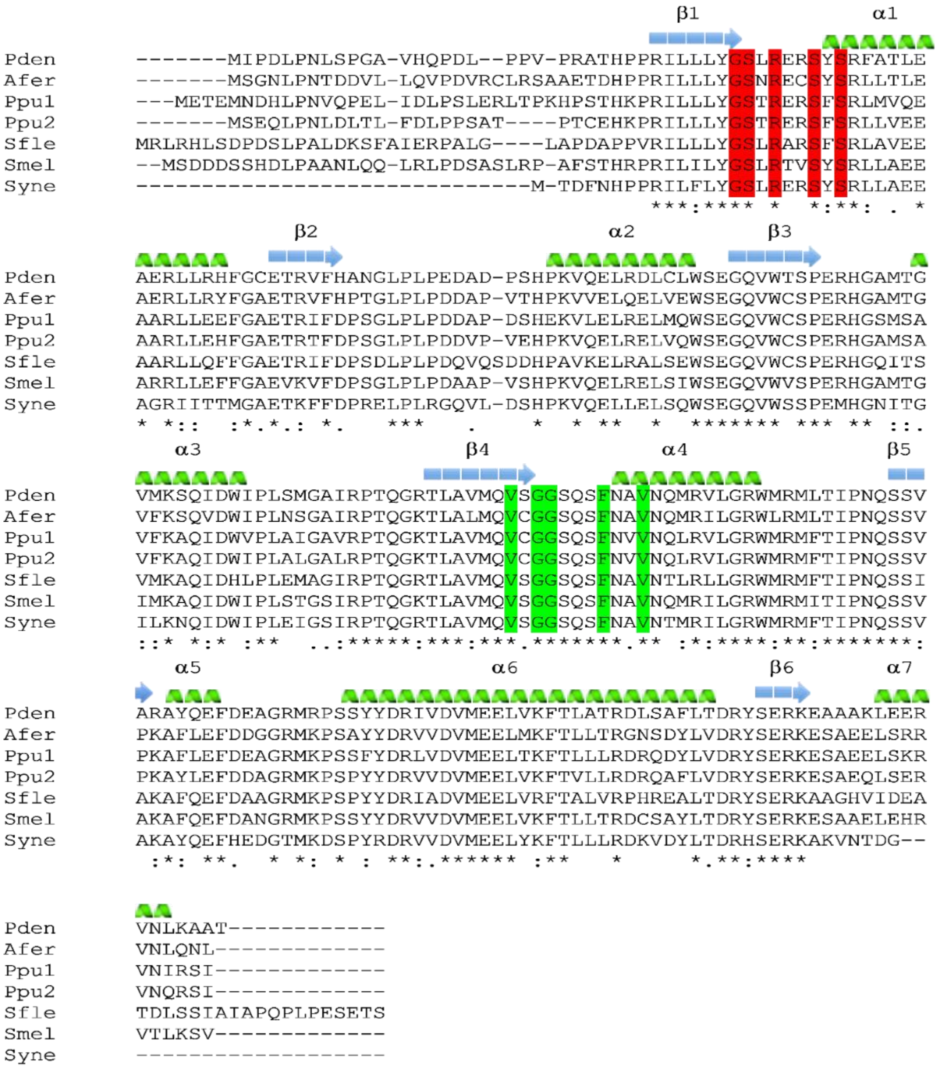

Figure S1. Multiple sequence alignment of *P. denitrificans* ArsH with homologous proteins.

Amino acid sequence alignment was generated with Clustal Omega [1], with the symbols below the alignment complete conservation (*), sites with strongly conserved properties (:), and sites with weakly conserved properties (.) The consensus motif for binding of the phosphate group of FMN [2] is shaded in red. The amino acid residues shaded in green match the consensus sequence described for $NADP^{+}$ binding proteins [3]. The UniProt accession codes for the ArsH proteins included here are: A1B6S0 (Pden, *Paracoccus denitrificans*), B7J950 (Afer, *Acidithiobacillus ferrooxidans*), Q88LK4 (Ppu1, *Pseudomonas putida*), Q88JD4 (Ppu2, *Pseudomonas putida*), Q7UC03 (Sfle, *Shigella flexneri*), Q92R45 (Smel, *Sinorhizobium meliloti*), P74312 (Syne, *Synechocystis* sp.).

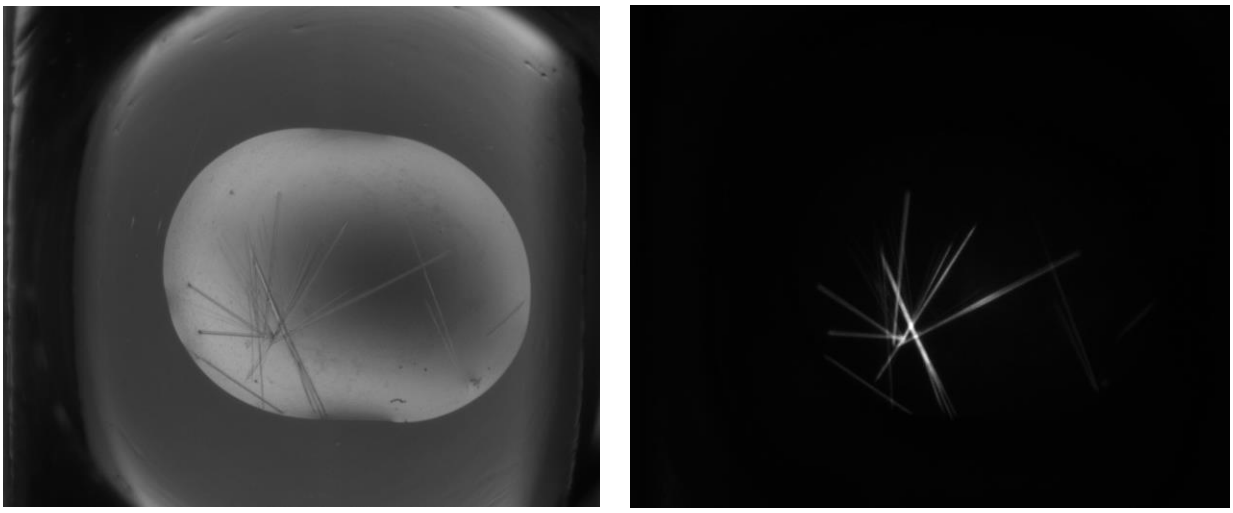

Figure S2. Crystals of *P. denitrificans* ArsH under VIS (left) and UV (right) illumination (Minstrel HT-UV + Gallery HT, Rigaku).

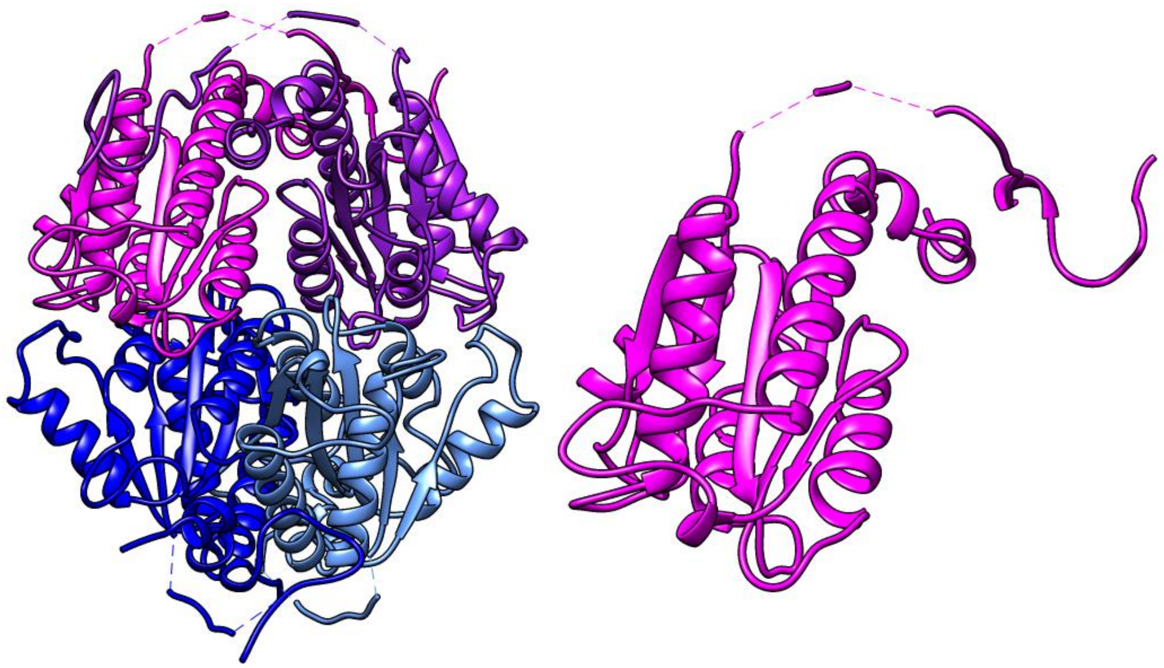

Figure S3. The crystal structure of *P. denitrificans* ArsH tetramer (left) and monomer (right)
The monomers are colored magenta (A), purple (B), blue (C) and cornflower blue (D). The missing parts of the structure are linked by dotted lines. The image was generated with Chimera 1.13.1.

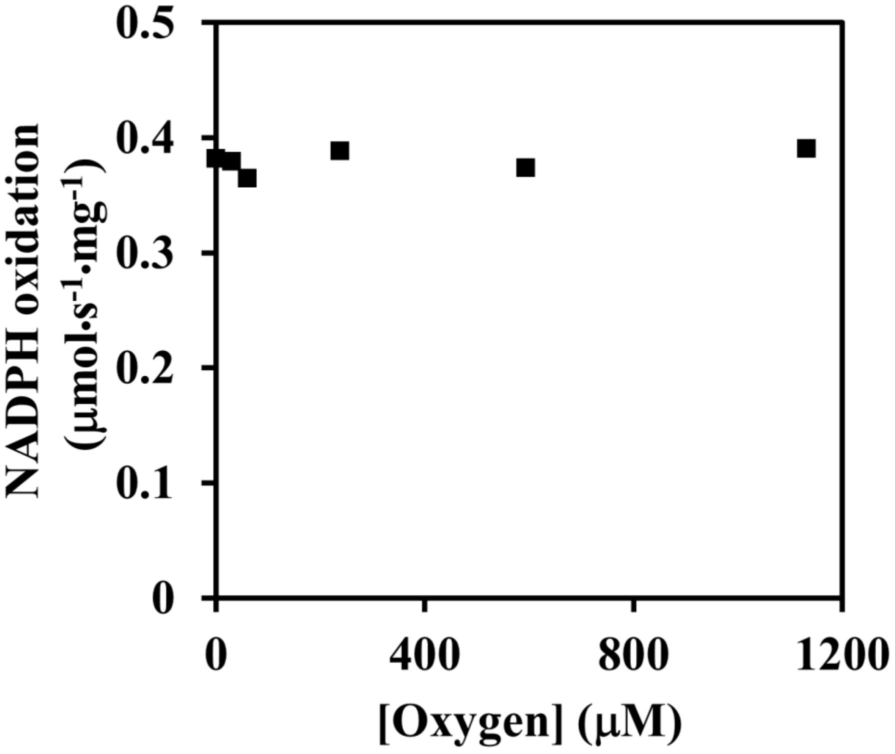

Figure S4. Dependence of the rate of NADPH oxidation, as catalyzed by ArsH on oxygen concentration. The reaction mixture (1 mL) contained 0.1 M sodium phosphate (pH 7.0, 30 °C), 0.15 mM NADPH and 0.05 mM FMN. The initial dissolved oxygen concentration was varied by mixing an O_2-saturated buffer solution and an N_2-saturated one. The saturated oxygen concentration equilibrated with 1 atm O_2 gas was taken to be 1.1 mM. The reaction was started by adding 205 nM ArsH.

```
AferArsR        MEPLQDPAQIVARLEALASPVRLEIFRLLVEQEPTGLVSGDIAEHLGQP-HNGISFHLKN
PdenArsR1       ----MEEQHALVGFAALSQETRLRIVRLLVKAGPEGMAAGAIGEALGGASTSRLSFHLTH
PdenArsR2       ----MDEQRALAGFAALSQETRLRIVRLLVKAGPEGMAAGAIGEALGGASTSRLSFHLTH
                     :  : :. : **:. .**.*.****:  * *:.:* *.* **    . :****.:

AferArsR        LQHAGLVTVQREGRYQRYRAAMPVVRALVAYLTENCCHGTRDCALSGET-RSPSVQEGNQ
PdenArsR1       LEHAGLIRSRREGRFIIYSAVFATLAGLIAFLMRDCCDGHPDICAPVAAALDCACDPTLT
PdenArsR2       LEQAGLIRSRREGRFIIYSAVFPTLAGLIAFLMRDCCDGHPEICAPVAAALSCSCEPGKD
                *::***:  :****:  * *.: .: .*:*:* .:**.*  : .    :  . : :

AferArsR        ----
PdenArsR1       REA-
PdenArsR2       ALHG
```

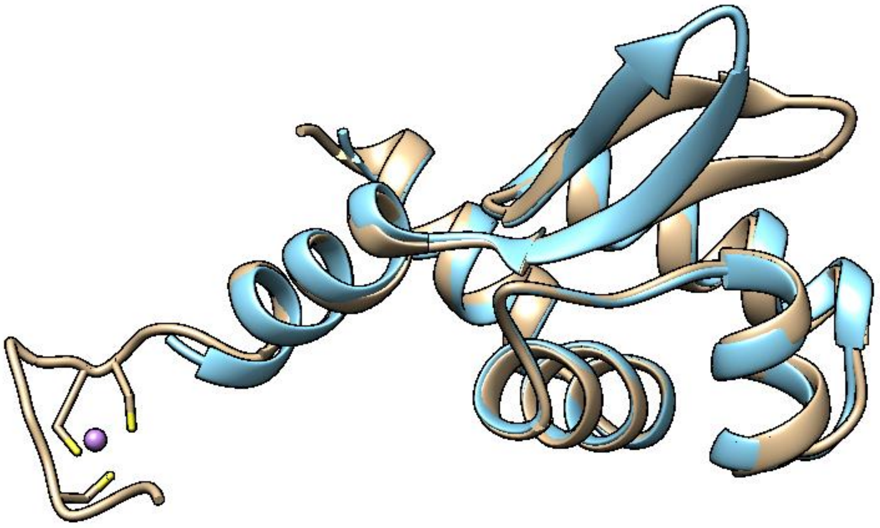

Figure S5. Sequence alignment and a model structure of the PdenArsR.

Upper part: Alignment of the amino acid sequences of the PdenArsR1 (Pden_3136) and PdenArsR2 (Pden_3342) proteins with the *Acidithiobacillus ferrooxidans* ArsR (Lferr_2480). Cysteine residues involved in binding arsenite to the *A. ferrooxidans* ArsR and cysteine residues of the *P. denitrificans* ArsRs are shaded in red.

Bottom part: A homology model structure of PdenArsR1 based on the x-ray structure of AferrArsR bound with arsenite (PDB ID: 6J05; [4]). The model was generated using Protein Homology/analogY Recognition Engine V 2.0 (Phyre2) [5]. 88 residues (74 % of the sequence) have been modeled with 99.9 % confidence. The incompleteness is due to low similarity between the model and template C-terminal sequences. The model structure (cyan colored) is superimposed onto the structure of the template (beige colored). The purple sphere depicts the bound trivalent arsenic ion. The image was generated with Chimera 1.13.1.

References

1. Madeira, F.; Park, Y.M.; Lee, J.; Buso, N.; Gur, T.; Madhusoodanan, N.; Basutkar, P.; Tivey, A.R.N.; Potter, S.C.; Finn, R.D.; et al. The EMBL-EBI search and sequence analysis tools APIs in 2019. *Nucleic Acids Res.* **2019**, *47*, W636-W641.
2. Agarwal, R.; Bonanno, J.B.; Burley, S.K.; Swaminathan, S. Structure determination of an FMN reductase from *Pseudomonas aeruginosa* PA01 using sulfur anomalous signal. *Acta Crystallogr. D Biol. Crystallogr.* **2006**, *62*, 383-391.
3. Hua, Y.H.; Wu, C.Y.; Sargsyan, K.; Lim, C. Sequence-motif detection of NAD(P)-binding proteins: discovery of a unique antibacterial drug target. *Sci. Rep.* **2014**, *4*, 6471.
4. Prabaharan, C.; Kandavelu, P.; Packianathan, C.; Rosen, B.P.; Thiyagarajana, S. Structures of two ArsR As(III)-responsive transcriptional repressors: Implications for the mechanism of derepression. *J. Struct. Biol.* **2019**, *207*, 209-217.
5. Kelley, L.A.; Mezulis, S.; Yates, C.M.; Wass, M.N.; Sternberg, M.J.E. The Phyre2 web portal for protein modeling, prediction and analysis. *Nat. Protoc.* **2015**, *10*, 845-858.
